# Peroxide of Hydrogen and Ozone—Their Antiseptic Properties

**Published:** 1890-12

**Authors:** 


(SELECTIONS.)

### PEROXIDE OF HYDROGEN AND OZONE—THEIR ANTISEPTIC PROPERTIES.

Read before the International Medical Congress, held at Berlin, Germany, on the 7th of August, 1890. Published by *Medical News*, of Philadelphia, October 25th, 1890. Pp. 416-418. By Dr. Paul Gibier, Director of the Pasteur Institute of New York.

GENTLEMEN—Since the discovery of Peroxide of Hydrogen by Thenard, in 1818, the therapeutical applications of this oxygenated compound seem to have been neglected both by the medical and surgical professions; and it is only in the last twenty years that a few bacteriologists have demonstrated the germicidal potency of this chemical.

Among the most elaborate reports on the use of this compound may be mentioned those of Paul Bert and Regnard, Baldy, Pean and Larrive.

Dr. Miguel places Peroxide of Hydrogen at the head of a long list of antiseptics, and close to the silver salts.

Dr. Bouchut has demonstrated the antiseptic action of Peroxide of Hydrogen, when applied to diptheritic exudations.

Prof. Nocart, of Alfort, attenuates the virulence of the symptomatic microbe of carbuncle, before he destroys it, by using the same antiseptic.

Dr. E. R. Squibb,* of Brooklyn, has also reported the satisfactory results which he obtained with Peroxide of Hydrogen in the treatment of infectious diseases.

Although the above-mentioned scientists have demonstrated by their experiments that Peroxide of Hydrogen is one of the most powerful destroyers of pathogenic microbes, its use in therapeutics has not been as extensive as it deserves to be.

In my opinion the reason for its not being in universal use is the difficulty of procuring it free from hurtful impurities. Another objection is the unstableness of the compound, which gives off nascent oxygen when brought in contact with organic substances.†

Besides the foregoing objections, the surgical instruments decompose the peroxide, hence, if an operation is to be performed, the surgeon uses some other antiseptic during the procedure, and is apt to continue the application of the same antiseptic in the subsequent dressings.

Nevertheless, the satisfactory results which I have obtained at the Pasteur Institute of New York, with Peroxide of Hydrogen, in the treatment of wounds resulting from deep bites, and those which I have observed at the French clinic of New York, in the treatment of phagedenic chancres, varicose ulcers, parasitic diseases of the skin, and also in the treatment of other affections caused by germs, justify me in adding my statement as to the value of the drug.

But, it is not from a clinical standpoint that I now direct attention to the antiseptic value of Peroxide of Hydrogen. What I now wish is merely to give a full report of the experiments which I have made on the effects of Peroxide of Hydrogen upon cultures of the following species of pathogenic microbes: Bacillus anthracis, bacillus pyocyaneous, the bacilli of typhoid fever, of Asiatic cholera, and of yellow fever, streptococus pyogenes, micro-bacillus prodigiosus, bacillus megaterium, and the bacillus of osteomyelitis.

The Peroxide of Hydrogen which I used was a 3 2 per cent.

---

* *Gaillard's Medical Journal*, March, 1889.

† The Peroxide of Hydrogen that I use is manufactured by Mr. Charles Marchand, of New York. This preparation is remarkable for its uniformity in strength, purity and stability.

solution, yielding fifteen times its volume of Oxygen; but this strength was reduced to about 1.5 per cent., corresponding to about eight volumes of Oxygen, by adding the fresh culture containing the microbe upon which I was experimenting. I have also experimented upon old cultures loaded with a large number of the spores of the bacillus anthracis. In all cases my experiments were made with a few cubic centimetres of culture in sterilized test-tubes, in order to obtain accurate results.

The destructive action of Peroxide of Hydrogen, even diluted in the above proportions, is almost instantaneous. After a contact of a few minutes, I have tried to cultivate the microbes which were submitted to the peroxide, but unsuccessfully, owing to the fact that the germs had been completely destroyed.

My next experiments were made on the hydrophobic virus in the following manner:

I mixed with sterilized water a small quantity of the medulla taken from a rabbit that had died of hydrophobia, and to this mixture added a small quantity of Peroxide of Hydrogen. Abundant effervescence took place, and, as soon as it ceased, having previously trephined a rabbit, I injected a large dose of the mixture under the dura mater. Slight effervescence immediately took place and lasted a few moments, but the animal was not more disturbed than when an injection of the ordinary virus is given. This rabbit is still alive, two months after the inoculation.

A second rabbit was inoculated with the same hydrophobic virus which had not been submitted to the action of the peroxide, and this animal died at the expiration of the eleventh day with the symptoms of hydrophobia.

I am now experimenting in the same manner upon the bacillus tuberculosis, and if I am not deceived in my expectation, I will be able to impart to the profesession some interesting results.

It is worthy of notice that water charged, under pressure, with fifteen times its volume of pure oxygen has not the antiseptic properties of Peroxide of Hydrogen. This is due to the fact that when the peroxide is decomposed nascent oxygen separates in that most active and potent of its conditions next to the condition, or allotropic form, known as "Ozone." Therefore it is not illogical to conclude that ozone is the active element of Peroxide of Hydrogen.

Although Peroxide of Hydrogen decomposes rapidly in the presence of organic substances, I have observed that its decomposition is checked to some extent by the addition of a sufficient quantity of glycerine; such a mixture, however, cannot be kept for a long time, owing to the slow, but constant formation of secondary products, having irritating properties.

Before concluding, I wish to call attention to a new oxygenated compound, or rather ozonized compound, which has been recently discovered, and called "Glycozone," by Mr. Marchand.

The Glycozone results from the reaction which takes place when glycerin is exposed to the action of ozone under pressūre—one volume of glycerin with fifteen volumes of ozone produces Glycerine.

By submitting the bacillus anthracis, pyocyaneous, prodigiosus, and megaterium to the action of Glycozone, they were almost immediately destroyed.

I have observed that the action of Glycozone upon the typhoid fever bacillus, and some other germs, is much slower than the influence of Peroxide of Hydrogen.

In the dressing of wounds, ulcers, etc., the antiseptic influence of Glycozone is rather slow if compared with that of Peroxide of Hydrogen, with which it may, however, be mixed at the time of using.

It has been demonstrated in Pasteur's laboratory that glycerin has no appreciable antiseptic influence upon the virus of hydrophobia; therefore, I mixed the virus of hydrophobia with glycerine, and at the expiration of several weeks all the animals which I inoculated with this mixture died with the symptoms of hydrophobia.

On the contrary, when glycerin has been combined with ozone to form Glycozone, the compound destroys the hydrophobic virus almost instantaneously.

Two months ago, a rabbit was inoculated with the hydrophobic virus, which had been submitted to the action of this new compound, and the animal is still alive.

I believe that the practitioner will meet with very satisfactory results with the use of Peroxide of Hydrogen for the following reasons:

1. This chemical seems to have no injurious effect upon animal cells.

2. It has a very energetic destructive action upon vegetable cells—microbes.

3. It has no toxic properties; five cubic centimetres injected beneath the skin of a guinea-pig do not produce any serious result, and it is also harmless when given by the mouth.

As an immediate conclusion resulting from my experiments, my opinion is, that Peroxide of Hydrogen should be used in the treatment of diseases caused by germs, if the microbian element is directly accessible; and it is particularly useful n the treatment of infectious diseases of the throat and mouth.

